# Retrospective Study of Clinico-Aetiological Factors of Chronic Urticaria Among Children Attending a Tertiary Care Paediatric Centre in Eastern Province of Sri Lanka

**DOI:** 10.7759/cureus.14848

**Published:** 2021-05-04

**Authors:** Vijayakumary Thadchanamoorthy, Kavinda Dayasiri, S Anputhasan

**Affiliations:** 1 Clinical Sciences Department, Faculty of Health Care Sciences, Eastern University, Batticaloa, LKA; 2 Internal Medicine-Pediatrics, Base Hospital, Mahaoya, LKA; 3 Paediatrics, Paediatric Professorial Unit, Teaching Hospital, Batticaloa, LKA

**Keywords:** chronic urticaria, eosinophilia, children, eastern province, sri lanka

## Abstract

Introduction*:* Chronic urticaria is one of the growing problems worldwide and the prevalence is increasing. Around 2% of children have been shown to be affected with chronic urticaria.

Objectives*:* To evaluate demographic, clinical and aetiological factors related to chronic urticaria in children and to assess investigations and treatment.

Method: A retrospective study was done from January 2018 to December 2019 on 40 children aged 1-14 years who presented with chronic urticaria to the paediatric clinic, University Paediatric Unit, Batticaloa Teaching Hospital. Detailed information including demographic factors, duration of illness, main reasons for clinic attendance, treatment received from the out-patient department, caregiver reported allergens, family history of atopy and complications such as secondary bacterial infection were retrieved from clinic-based records. Information regarding laboratory investigations was also retrieved. Data were analysed using SPSS version 19.0 (IBM Corp., Armonk, NY).

Results*:* Forty children were available for the analysis. The most common age category was four to eight years and 25 (62.5%) were females. Seventy percent of children had urticaria for one or more years. Thirty-six (90%) children had a family history of allergies such as allergic rhinitis, eczema, food allergies, medication allergies and bronchial asthma. The common precipitating factors were foods in 12 (30%) cases and insect bites in 10 (25%) cases. The main reasons for seeking medical advice were severe itchiness in 40 (100%) and sleep disturbances in 24 (60%). Only a limited number of investigations had been performed and they included white cell counts (differential eosinophil counts) and thyroid functions. Almost all had normal thyroid functions whilst 45% had eosinophilia.

Conclusions*: *The majority of children with chronic urticaria had symptoms for over the one-year duration and reported a family history of allergy. All children reported severe itchiness as the main symptom and sleep was disturbed in the majority. The common precipitating factors were food and insect bites.

## Introduction

Urticaria is a skin disorder that presents as a rash comprising pale red, raised and itchy lumps [1]. The lesions usually disappear within a few hours and reappear at different sites over time [2] causing anxiety in most parents. These lesions occur due to vascular reactions of multiple etiologies [3]. It is clearly understood that the lesions are induced by various vaso-active substances including histamine, kinins and prostaglandins [2]. Acute urticaria typically lasts for less than six weeks whilst chronic urticaria persists for more than six weeks [4]. Acute urticaria does not need investigation and resolves within six weeks mostly with symptomatic treatment [5]. However, chronic urticaria often needs investigation and follow-up due to its association with other auto-immune conditions and underlying etiological factors [5]. Chronic urticaria is mostly idiopathic although approximately 35-40% of cases with chronic idiopathic urticaria could have autoimmunity as an underlying etiological factor [6]. Viral infections also play a role in chronic urticaria [7].

Chronic urticaria is one of the growing problems worldwide and the prevalence is increasing [8]. The disease is more common in Asian populations as compared to populations in Europe and Northern America [8]. Approximately, 2% of children have been shown to be affected with chronic urticaria [9]. In addition to causing physical symptoms, the disease can have a potentially significant psychological impact due to cosmetic troubles, parental anxiety, sleep disturbances, restriction of food choices and overall reduced quality of life [10,11].

The aim of this study was to evaluate demographic, clinical and aetiological factors related to chronic urticaria in children and to assess investigations and treatment.

## Materials and methods

A retrospective hospital-based study was conducted among children who attended the paediatric clinic, University Paediatric Unit, Batticaloa Teaching Hospital over two years from January 2018 to December 2019. Forty patients who were diagnosed with chronic urticaria (urticaria lasting more than six weeks) between ages one to fourteen years were analysed. In selecting children for the study, the diagnosis was verified for accuracy and only children who were diagnosed by the consultant paediatrician were recruited. Children with doubtful diagnoses were ruled out from the study.

Detailed information including demographic factors, duration of illness, main reasons for clinic attendance, treatment received from the out-patient department, main allergens, family history of atopy and complications such as secondary bacterial infection were retrieved from clinic-based records. Information regarding laboratory investigations was also retrieved. Data were analysed using the SPSS version. 19.0 (IBM Corp., Armonk, NY).

Eosinophil counts exceeding 0.5 × 10^9^/l (500/μL) were considered as eosinophilia because the normal range is 0.1-0.5 × 10^9^/l.

As it was a retrospective study, no ethical clearance was obtained. However, permission was obtained from the Director, Teaching Hospital Batticaloa to retrieve clinic-based medical records.

## Results

Forty children were recruited to the study comprising 25 (62.5%) females and 15 (37.5%) males. Twenty-eight (70%) children were below eight years of age. Table 1 shows the distribution of children based on the age category. Twelve children (30%) had chronic urticaria for less than a year whilst ten children (25%) had chronic urticaria for over two years.

**Table 1:** Age distribution of the study sample

Age group (years)	Female	Male	Total
1 to <4	06	04	10
4 to <8	13	05	18
8 to <12	04	04	08
12 to 14	02	02	04

There were several reasons which made parents and children seek medical attention and the reasons included severe itchiness, sleep disturbances, abdominal pain, vomiting and loose stools, fever and urticaria, and cosmetic problems. All children complained of severe itchiness as a primary reason for seeking medical attention. The frequency of these factors is shown in Table 2.

**Table 2:** Reason for the first presentation to the clinic

Reason for the first presentation	Number (%)
Severe itchiness	40 (100)
Sleep disturbances	25 (62.5)
Abdominal pain	10 (25.0)
Vomiting and loose stools	08 (20.0)
Cosmetic problem	06 (15.0)
Fever	04 (10.0)
Breathing difficulty	02 (05.0)

Thirty-six (90%) patients had a family history of atopy. The reported inciting agents causing chronic urticaria were foods, insect stings and medications, whilst the aetiology was unknown in some patients. The frequency of precipitating factors is shown in Table 3.

**Table 3:** Frequency of precipitating factors for chronic urticaria

Precipitating factor	Number (%)
Insect bites	12 (30.0)
Foods	10 (25.0)
Both foods and insect bites	03 (07.5)
Medications	08 (20.0)
Unknown aetiology	07 (17.5)

Insect bites that led to chronic urticaria included bites by fleas, ants and mosquitoes. The foods which were identified as precipitating chronic urticaria based on clinical history included tomatoes, brinjal, cashew, seafood and pine-apple. Medications that were implicated in mounting chronic urticarial reactions were paracetamol, non-steroidal anti-inflammatory drugs and antibiotics. Some children had chronic urticaria following exposure to multiple foods and multiple etiologies. In seven children, the precipitating factors were unknown.

Only limited investigations had been performed. Eosinophilia was present in 45% of children with chronic urticaria and none had abnormal thyroid function tests. All children had treatment at the outpatients' department and 10 (25%) children needed review by the consultant dermatologist for further evaluation and management.

## Discussion

Urticaria is one of the common medical problems in children [12,13]. Urticaria in most children is a self-resolving, brief illness that lasts for several days and leaves no dermatological stigmata over long term. However, in up to 5% of children with urticaria, lesions can last for more than six weeks leading to chronic urticaria [3,14,15]. Chronic urticaria often recurs [5] causing unacceptable discomfort to children and anxiety in parents. Although making the diagnosis of chronic urticaria is not difficult, determining the underlying etiology is often challenging. Rarely, it might be a manifestation of a hidden sinister disorder [16]. Chronic urticaria can potentially create a diagnostic challenge to paediatricians whenever the etiology is unknown. 

Literature regarding chronic urticaria among children in South Asia is limited [3]. A study conducted in a tertiary center in India reported that approximately 80% of children attending an urticaria clinic had chronic urticaria with over 50% having no identified etiology [17]. In the current study, chronic urticaria was more common in children below the age of eight years (70%) compared to those who were between 9 and 14 years (30%). The study further showed that female children were predominantly affected. A systematic review on the global epidemiology of chronic urticaria reported that women were predominantly affected, but in patients who were less than 15 years, the gender difference was less apparent [8]. The predominant prevalence of female patients has been reported in several other studies [18-20]. One reason for female predominance could be that 35-40% of chronic urticaria have an autoimmune origin [4]. In contrast, cholinergic urticaria had been slightly more common in males compared to females in another study [21].

A study of adults with chronic urticaria showed that it was common in all age groups with no differences seen based on economic status, geographical location, and size of city [22]. However, Asian studies have shown that moving to a new residence and living in a high-income family were risk factors for chronic urticaria [9]. The current study was based on a predominantly rural population; however, precise risk factors could not be determined given the retrospective nature of the study. In the current study, 70% of children had urticarial symptoms for at least one-year duration. However, a study from South Korea reported that most children had symptoms for less than one-year duration [21]. This could be due to the fact that most children in the current study were initially managed by general practitioners before they were referred to paediatricians.

The current study had to rely on clinical history-based information to determine the potential etiological factors given the lack of specific investigation facilities in the study setting and the retrospective nature of the study. Approximately 90% of the patients had a positive family history of atopy including manifestations of one or a combination of conjunctivitis, rhinitis, eczema and bronchial asthma suggesting the possible multi-factorial nature of its causation. This study showed precipitating factors in 33 (82.5%) of children with chronic urticaria. These factors included insect stings, foods, both insect stings and foods, and medications. However, in seven children etiology could not be determined based on clinical history. In studies conducted to explore the etiological factors for chronic urticaria, the etiology was unknown in a substantial proportion [23-25].

The reasons for attendance of consultant paediatrician led clinic were also analysed in the current study. The study showed that all patients had severe itching while sleep disturbances were present in 60%. Other reasons included systemic symptoms such as fever, abdominal pain, breathing difficulty, vomiting and loose stools. Cosmetic problems were also a reason in six children. Studies have reported a higher prevalence of sleep disturbance, anxiety, and depressive symptoms with a significant negative impact on work and leisure activities [10,11] in patients with chronic urticaria. Secondary bacterial infection was found only in 30% of children with chronic urticaria in the current study and they needed treatment with oral antibiotics.

Investigation of children with chronic urticaria was limited. Eosinophilia was found in 45% of patients and thyroid function had been normal in all patients. However, specific investigations such as anti-nuclear antibodies, anti-thyroperoxidase, and IgE levels had been performed inconsistently and only in a very limited number of children. Autologous serum skin testing had not been performed in any child due to the lack of availability of the test in the study setting. It is therefore important to widen the facilities for the investigation of these children so that children and parents can be offered more accurate information regarding etiology and precipitating factors.

The study has several limitations. The retrospective nature of the study limited the exploration of specific risk factors for chronic urticaria in the study sample. The precipitating factors were determined based on clinical history rather than specific skin prick allergy testing due to the lack of availability of this investigation in the study setting. However, the investigators retrieved only the information which could be reliably gathered from medical records such as a limited number of demographic factors, reasons for the first presentation and history-based etiological and precipitating factors.

The study brings to light that chronic urticaria in children, although not very common, can be recurrent over two years in up to 25% of children and potentially distressing to both children and parents due to recurrent severe itchiness, disturbed sleep, cosmetic problems and a range of multi-systemic physical symptoms. Studies are needed to assess the quality of life and physical and psychological impact of chronic urticaria in the study population.

## Conclusions

The majority of children with chronic urticaria had symptoms for over the one-year duration and reported a family history of allergy. All children reported severe itchiness as the main symptom and sleep was disturbed in the majority. The common precipitating factors were food and insect bites.
